# Stressed and overworked? A cross-sectional study of the working situation of urban and rural general practitioners in Austria in the framework of the QUALICOPC project

**DOI:** 10.3325/cmj.2015.56.366

**Published:** 2015-08

**Authors:** Kathryn Hoffmann, Silvia Wojczewski, Aaron George, Willemijn L. A. Schäfer, Manfred Maier

**Affiliations:** 1 Department of General Practice, Center for Public Health, Medical University of Vienna, Vienna, Austria; 2 Department of Community and Family Medicine, Duke Medical Center, Durham, NC, USA; 3 NIVEL – the Netherlands Institute for Health Services Research, Utrecht, the Netherlands

## Abstract

**Aim:**

To assess the workload of general practitioners (GPs) in Austria, with a focus on identifying the differences between GPs working in urban and rural areas.

**Methods:**

Within the framework of the Quality and Costs of Primary Care in Europe (QUALICOPC) study, data were collected from a stratified sample of GPs using a standardized questionnaire between November 2011 and May 2012. Data analysis included descriptive statistics and regression analysis.

**Results:**

The analysis included data from 173 GPs. GPs in rural areas reported an average of 49.3 working hours per week, plus 23.7 on-call duties per 3 months and 26.2 out-of-office care services per week. Compared to GPs working in urban areas, even in the fully adjusted regression model, rural GPs had significantly more working hours (B 7.00; *P* = 0.002) and on-call duties (B 18.91; *P* < 0.001). 65.8% of all GPs perceived their level of stress as high and 84.6% felt they were required to do unnecessary administrative work.

**Conclusion:**

Our findings show a high workload among Austrian GPs, particularly those working in rural areas. Since physicians show a diminishing interest to work as GPs, there is an imperative to improve this situation.

General practitioners’ (GPs) workload is an increasingly important research topic as strengthening the primary care sector has become one of the key health care challenges. In many countries numbers of GPs are dropping (1-4) and high workload is a prominent contributing factor to physicians’ decreased interest to work as GPs (5-10), as well as to stress and time pressures in this population. This is all especially pronounced in rural areas. As shortages continue and worsen, provision of adequate medical services to growing and aging population could become a serious concern, especially in the midst of shorter consultation times and decreasing quality of communication and care (11,12).

Workload can be viewed as objective and subjective. Objective workload is measured as the amount of work, time involved, or the frequency at which activities take place (13,14), while subjective workload is viewed as the experienced workload by a given provider or group (13). Objective workload is influenced by the list size and the practice workforce composition and these in turn impact job satisfaction (13). Yet, until now, research on GPs’ workload has been mainly conducted in health care systems where GPs have a set patient list and are paid by a capitation system. In fee-for-service systems, particularly those where GPs have no specific patient list or gatekeeping function, the workload issue was experienced as having less impact on job satisfaction (13). It follows that GPs working in such systems garner greater financial incentives for working longer hours or seeing more patients, which likely influences job satisfaction (6,9,13-15). Meanwhile, both subjective and objective workload have rarely been evaluated within the common and rural GP practice setting.

Austria is a Bismarck-system country without gatekeeping or list system, where GPs are self-employed and are remunerated by a mixed payment model that is predominantly fee-for-service (16). The Bismarck model uses an insurance system and is financed jointly by employers and employees through payroll deduction. Bismarck-type health insurance plans do not make a profit and must include all citizens (17).

Austria maintains a stable flow of medical students through its universities and has one of Europe’s highest number of medical graduates (18). Despite this, the number of graduates interested in GP training is steadily decreasing (19). It is expected that by 2030 the overall need for GPs in Austria will increase by 22% (19). Also, within the next ten years about 30% of all practicing GPs will reach retirement age (20), and the number of young physicians to take their place is much lower. The decline in interest may be partly attributable to the lack of adequate under- and post-graduate exposure, education, and training in general practice (21-24). This is likely further challenged by the lack of academic recognition of general practice as a specialty (16,25). Recent studies suggest that the reasons for decreased interest in GP training are rooted in fears about high workload, especially in rural areas, the potential for lower income than in other specializations, and greater social constraints (5,8-10).

Against this background, and in the framework of the Quality and Costs of Primary Care in Europe (QUALICOPC) questionnaire study (26,27), our analysis had three aims: first, we evaluated the objective workload of Austrian GPs in terms of working hours, patient contacts (in and outside the office), consultation time, and involvement in out-of-hours services. Second, we examined the influence of practice workforce composition (solo-practice vs group practice) on the physicians’ workload. Third, we assessed the subjective work satisfaction and the influence of objective workload pattern on work satisfaction.

## Methods

### Design

The data were collected as part of the European QUALICOPC project, a cross-sectional study that compared the quality, fairness, and cost of primary care systems in Europe, Canada, Australia, and New Zealand (26,27). Between October 2011 and May 2012, Austrian GPs were recruited to complete a questionnaire on their work situation. The study was designed in accordance with the STROBE statement for cross-sectional studies (28) and approved by the ethics committee of the Medical University in Vienna (EC N° 808/2011).

### Recruitment

In Austria, the standardized and translated QUALICOPC questionnaire was sent to GPs interested in participating in this study. They were invited to participate in several ways: via the email list server of the voluntary Society of General Practitioners, email addresses posted on the websites of the federal medical associations, and through personal contacts. In all, we attempted to invite 3050 of 6527 GPs working in the ambulatory sector in 2010 by electronic means, and of these 1828 had a valid email address. To these 1828 GPs, the questionnaire was sent electronically together with a short description of the study and the informed consent form, with three subsequent reminders. The aim was to recruit 180 doctors, representing all nine federal states, both sexes, and different age groups, as well as GPs with and without a contract with public social health insurance companies. The inclusion criteria were that the GP had to have an office in Austria and that only one GP per office would be surveyed. An additional inclusion criterion for this analysis was to include only GPs with a contract with public social health insurance companies. This was done because private GPs differ from contracted GPs with regard to the average number of patients per day, working time (they do not have an obligatory minimum working time), and number of on-call duties. Before inclusion, all GPs completed the informed consent form. Altogether, 196 GPs expressed interest (return rate: 10.7%), but 12 GPs did not participate because of time concerns. Our final sample included 184 GPs and was comparable to the national sample with regard to sex, age, office location, and solo- vs group practice distribution (20).

### Questionnaire

The questionnaire contained 60 questions, was the result of a multi-step developmental approach, and was based on a pilot survey (27). It was translated into the languages of the participating countries by the national coordinators. In Austria, readability and feasibility of the translated questionnaire was checked by a group of medical students and colleagues (GPs and medical anthropologists) and the questionnaire was back-translated into English.

*Dependent variables.* Objective workload was assessed by the number of working hours per week as GP (excluding additional jobs, on-call duties, and out-of-hours services), hours spent per week on direct patient care, number of patients contacts per day, number of on-call duties during evenings/nights/weekend days in the past three months, number of home visits and visits to retirement homes per week, and average consultation time. Average consultation time was assessed with the question: “How long does a regular patient consultation in your office usually take?”. Subjective work satisfaction was assessed with the items: “I feel that some parts of my work do not really make sense,” “My work still interests me as much as it ever did,” “My work is overloaded with unnecessary administrative detail,” “I have too much stress in my current job,” “In my work there is a good balance between effort and reward.” The answers were strongly agree, agree, disagree, and strongly disagree. These answer categories were dichotomized into agree (strongly agree and agree) and disagree (disagree and strongly disagree).

*Independent variables.* The workload-influencing variables included practice workforce composition and list size (13). These variables were assessed with the questions “Do you work alone or in a shared accommodation with one or more GPs and/or medical specialists?” with the answer options “alone” or “not alone” and with the question “What is the estimated size of your practice population?”. Another variable relevant for this analysis was location of the GP office (big city, suburbs, small town, mixed urban-rural, rural). Additionally, we assessed the age and sex of the GPs.

### Data analysis

First, the variable location of office was clustered into urban (big city, suburban), intermediate (small town and mixed), and rural areas. Next, the relationship between demographic variables of the GPs (age and sex) and all other dependent and independent variables in relation to the location was described by descriptive statistical methods. Subgroup analyses were conducted by means of contingency table tests. To test the differences between the groups, Fisher´s exact test or ANOVA one-way test, including post-hoc Tukey test, was applied after testing for normal distribution. If independency could not be proven by Fisher´s exact test, z-test, including the Bonferroni method for multiple testing, was applied to determine which sub-groups were dependent.

Multivariable mixed linear regression models were used to assess the association of the location variable and other variables possibly influencing workload on the objective workload. Variables analyzed were the number of working hours per week as GP (excluding additional jobs, number of on-call duties, and out-of-hours services), hours spent on direct patient care per week, number of on-call duties during evenings/nights/weekend days in the past three months, and number of home visits plus number of visits to retirement homes per week. Correlations were calculated between these variables to identify strong correlations and exclude those from the regression model to avoid collinearity. Thus, the “hours spent per week on direct patient care” variable was excluded as it correlated strongly with the number of working hours. All other variables were taken into the model simultaneously. The location of GP office variable was entered into the model as a categorical variable, with urban location as reference group for the intermediate and rural location groups, respectively. The adjusted R˛ is presented as a measure of model-fit. A similar multivariable regression model was used for the subjective work satisfaction variables, first, for the single items and for the satisfaction score after having calculated a sum score for the five questions related to work satisfaction. The answers expressing most work satisfaction received one point and the answers expressing least work satisfaction received four points. The significance level for all calculations was *P* < 0.05, the confidence interval 95%. SPSS Statistics 22.0 (IBM, Armonk, NY, USA) was used for all analyses.

## Results

Of 184 GPs available for the study, 11 were excluded because they did not have a contract with any public social health insurance company. The final analysis included the remaining 173 GPs, 28.3% of whom (n = 49) were women. There was no significant difference in practice location between men (urban 35.5% [n = 44], intermediate 32.3% [n = 40], rural 32.3.4% [n = 40]) and women (urban 44.9% [n = 22], intermediate 32.7% [n = 16], rural 22.4% [n = 11], *P* = 0.374)

### Significantly higher objective workload in intermediate and rural areas

Significantly higher workload was observed in intermediate and rural than in urban GP offices (*P* < 0.001). This was the case for nearly all objective workload variables, but was not observed in the workload-influencing variables. The mean and interquartile range for the estimated size of the practice population was 3768.9 (600-18,920) for urban GPs, 3659.3 (800-17,000) for intermediate area GPs, and 3288.4 (590-20,000) for rural GPs (Table 1).

**Table 1:** Demographics and the objective workload variables in relation to office location

Variable	Urban (n = 66)	Intermediate (n = 56)	Rural (n = 51)	P*
Sex, % (n)				
female (n = 49)	34.9 (22)	28.6 (16)	22.9 (11)	0.391*
male (n = 124)	65.1 (44)	71.4 (40)	77.1 (40)	
GP practice, % (n)				
solo-practice (n = 153)	92.2 (59)	83.9 (47)	93.8 (47)	0.311*
group-practice (n = 18)	7.8 (5)	16.1 (9)	7.8 (4)	
Mean age (years), mean (SD)	55.4 (7.0)	54.1 (7.9)	54.4 (6.6)	0.616^†^
Number of working hours as GP, mean (SD)^ǁ^	40.7 (12.6)^‡^	45.3 (11.7)^‡,§^	49.4 (9.6)^§^	<0.001^†^
Hours spent per week on direct patient care, mean (SD)	33.7 (13.1)^‡^	36.7 (11.4)^‡,§^	40.3 (10.6)^§^	0.019^†^
Number of on-call duties in the past 3 months, mean (SD)				
evening	1.8 (4.7)^‡^	7.3 (15.8)^‡,§^	7.9 (12.1)^§^	0.023^†^
night	2.1 (5.3)^‡^	9.5 (15.1)^§^	10.5 (9.0)^§^	<0.001^†^
weekend	1.0 (2.3)^‡^	3.4 (2.1)^§^	5.3 (7.1)^§^	<0.001^†^
total	4.8 (11.2)^‡^	20.2 (29.6)^§^	23.7 (21.8)^§^	<0.001^†^
Number of out-of-office visits, mean (SD)				
home visits	13.5 (10.4)	16.4 (10.5)	17.4 (11.6)	0.137^†^
retirement homes	6.9 (12.0)^‡^	12.5 (11.0)^§^	8.8 (9.8)^‡,§^	0.023^†^
total	20.4 (18.6)^‡^	29.0 (18.8)^§^	26.3 (16.9)^‡,§^	0.035^†^
Number of patient contacts per day, mean (SD)				
face-to-face	45.8 (20.2)^‡^	56.4 (18.9)^§^	49.1 (19.1)^‡ §^	0.013^†^
by phone	11.1 (10.3)	11.4 (12.3)	10.3 (9.1)	0.870^†^
by email	1.7 (2.9)	2.2 (4.9)	0.8 (2.0)	0.118^†^
Estimated size of practice population, mean (SD)	3323.6 (2,135.1)	3407.6 (2318.3)	2,925.1 (2208.7)	0.520^†^
Average consultation time, mean (SD)	11.4 (14.8)	9.1 (3.4)	8.7 (3.6)	0.265^†^

*Fisher exact test including z-test.

†ANOVA one-way including Post-Hoc Tukey test.

‡§The superscript refers to within location (row) pairwise comparisons. Groups with the same superscript are not significantly different from each other at a significance level of *P* < 0.05.

ǁwithout on-call duties and out-of hours services)/week.

With regard to on-call duties, 89.4% of urban GPs, 55.4% of intermediate area GPs, and 58.3% of rural GPs worked no evening-shifts in the three months prior to the survey (*P* < 0.05 for rural and intermediate GPs compared to urban GPs). Similar results were reported for night-shifts, with 82.1% of urban GPs, 32.1% of intermediate area GPs, and 25.0% of rural GPs working no night-shifts (*P* < 0.05 for all). Also, 62.6% of urban GPs, 12.5% of intermediate area GPs, and 4.2% of rural GPs did not work weekend-shifts (*P* < 0.05 for all).

### Office location was significantly associated with workload

Office location was significantly associated with workload: in the adjusted multiple linear regression model rural GPs worked about seven hours more (B = 7.00, *P* = 0.002) than their urban colleagues. This was additionally the case with the number of out-of-office services per week (B = 0.20, *P* < 0.001) and the estimated size of the practice population (B = 0.002, *P* = 0.049). Sex, age, and practice composition were not associated with workload (Table 2). In addition, rural compared to urban location was significantly associated with the total number of on-call duties per three months (B = 18.91, *P* < 0.001).

**Table 2:** Mixed linear regression models for the general practitioner’s (GP) workload.

Coefficient: working hours per week as GP without on-call duties and out-of-hours services												
				Coefficient		*P*				95% confidence interval		
Intercept				41.90		<0.001				26.53-57.28		
Age (years)				-0.15		0.260				-0.42-0.11		
Sex												
male				2.43		0.219				-1.46-6.31		
female				0								
Estimated size of practice population			0.002		<0.049				0.001-0.002		
Average consultation time (min)			0.04		0.666				-0.40-0.22		
Total number of out-of-office visits per week			0.20		<0.001				0.10-0.30		
Place of practice location (reference: urban location)												
urban				0								
intermediate				1.24		0.558				-2.94-5.42		
rural				7.00		0.002				2.71-11.28		
Practice workforce composition												
solo practice				0								
group practice				-2.07		0.457				-7.57-3.42		
**Corrected R˛**				**0.219**								
**Coefficient: Total number of on-call duties per three months**												
Intercept	20.66						0.235	-13.57-54.88				
Age (years)	-0.36						0.222	-0.94-0.22				
Sex												
male	6.24						0.155	-2.40-14.88				
female	0											
Estimated size of practice population		<0.001					0.642	-0.001-0.001		
Place of practice location										
urban		0								
intermediate		15.85					0.001	6.49-25.21		
rural		18.91					<0.001	9.25-28.58		
Practice workforce composition										
solo practice		0								
group practice		-0.56					0.930	-13.17-12.05		
**Corrected R˛**		**0.113**								

### Overall work satisfaction was low

No significant differences were found between work satisfaction variables and the location stratified for sex. On average, 84.6% of GPs responded that their work was overloaded with unnecessary administrative detail, 65.8% that they experienced too much stress at work, and 35.7% that there was a good balance between effort and reward in work. On the other hand, 91.3% of GPs stated that their work still interested them as much as ever (Table 3).

**Table 3:** Work satisfaction variables in relation to the location variable

Variable, % (n)	Urban (n = 66)	Intermediate (n = 56)	Rural (n = 51)	P*
I feel that some parts of my work do not really make sense (agree)				
all	25.8 (17)	37.5 (21)	17.6 (9)	0.071
men	27.3 (12)	32.5 (13)	17.5 (7)	0.293
women	22.7 (5)	50.0 (8)	18.2 (2)	0.131
My work still interests me as much as it ever did (agree)				
all	89.4 (59)	87.5 (49)	96.1 (49)	0.273
men	84.1 (37)	85.0 (34)	95.0 (38)	0.270
women	100.0 (22)	93.8 (15)	100.0 (11)	0.349
My work is overloaded with unnecessary administrative detail (agree)				
all	83.3 (55)	82.1 (46)	86.3 (44)	0.837
men	81.8 (36)	82.5 (33)	85.0 (34)	0.955
women	86.4 (19)	81.3 (13)	90.9 (10)	0.775
I have too much stress in my current job (agree)				
all	59.1 (39)	75.0 (42)	60.8 (31)	0.147
men	56.8 (25)	72.5 (29)	65.0 (26)	0.354
women	63.6 (14)	81.3 (13)	45.5 (5)	0.165
In my work there is a good balance between effort and reward (agree)				
all	34.8 (22)	30.9 (17)	43.8 (22)	0.396
men	37.2 (16)	38.5 (15)	45.0 (18)	0.758
women	27.3 (6)	12.5 (2)	36.4 (4)	0.337

*Fisher exact test including z-test.

The work satisfaction score had a minimum of seven points (five points being very satisfied) and a maximum of 20 points (very dissatisfied), with a mean of 12.4 points (standard deviation, 2.6 points). The multivariable regression model for the satisfaction score resulted in no significant associations for any of the objective workload or demographic variables with the work satisfaction score (age: regression coefficient [B] = 0.003, *P* = 0.923; female: B = 0.06, *P* = 0.905; working hours per week: B = 0.03, *P* = 0.222; estimated size of practice population: B<0.001, *P* = 0.066; total number of out-of-office visits per week: B = 0.01, *P* = 0.531; total number of on-call duties per three months: B = 0.01, *P* = 0.581; rural location compared to urban location: B = 1.59, *P* = 0.175). However, when we ran the same regression model for the single work satisfaction variables separately, it was demonstrated that a GP experienced significantly increased stress levels in conjunction with more work hours (B = 0.02, *P* = 0.010) and significantly higher levels of dissatisfaction with administrative work in conjunction with a higher estimated size of the practice population (B = 0.01, *P* = 0.002) and higher age (B = 0.02, *P* = 0.49). No association with any other independent or control variable for the individual work satisfaction variables was found.

## Discussion

This study demonstrates that GPs with practices in rural regions in Austria have a significantly higher workload than their colleagues in urban areas. Rural GPs worked more than an entire full working-day each week than their colleagues in bigger cities. The mean workload of Austrian GPs, without side-jobs and on-call duties (mean 45.1 hours) is comparable to the workload of GPs in other industrialized and wealthy countries, eg, Germany (50.8 hours) (8), France (48.6 hours), USA (47.6 hours), UK (42.2 hours), and Australia (40.5 hours) (6).

When it comes to on call-duties, rural GPs reported almost five times more on call-duties per three months than their urban colleagues, with the largest discrepancy observed in the number of night- and weekend-shifts. These findings are supported by studies from Germany and Canada, which also showed significantly more working hours for rural GPs (8,15). One reason for the differences in the on-call duty frequency is likely the distinct organization of emergency services in rural and urban areas in Austria (16). In larger cities, the local government organizes the out-of-hours service for the ambulatory sector and each physician working in the ambulatory sector (GPs and all other disciplines) participates on a voluntary basis, independent from the contractor status with the social health insurance companies. These positions are often filled with young physicians without contractor status who want to earn extra money. In addition to this service, in the capital Vienna, for example, the majority of the 27 hospitals provide emergency department services, which can be accessed at any time for nearly any complaint, as Austria is a country lacking a gatekeeping system (29). In rural areas, it is up to the GPs to organize the out-of-hour services in primary care on a rotational basis. With a declining number of GPs in rural areas, this becomes a vicious cycle as the decreasing number of GPs creates an increasing number of on-call duties. The future portends worsening discrepancies, as young trainees voice diminishing interest in working in rural general practice. Additionally, GPs spent about eight hours per week on other tasks aside from direct patient care. Since approximately 84.5% of all GPs acknowledged high overall dissatisfaction with administrative work, we assume that additional hours assigned for these tasks may further discourage young physicians from becoming GPs.

The number of out-of-office care services per week (visits to retirement homes and number of home visits) is high for all GPs, with 20.4 instances of such care services in urban settings, compared with 26.3 instances in rural areas. This represents one of the highest rates of out-of-office services in Europe (30). Additionally, the number of personal patient contacts per day was high, with average of 50.4 contacts per day, as well as the estimated average practice population of 3218.8 per GP. Compared to other countries, Austria, along with Germany, has the greatest number of patient contacts per day (6). One reason for this, as for the high number of out-of-office services per week, is likely related to the mixed reimbursement model, with fee-for-service as the predominant financing system in primary care (2,31).

Surprisingly, the higher workload of GPs working in intermediate and rural areas is not reflected in an overall lower work satisfaction compared to the GPs working in bigger cities. All three groups were ambivalent on work satisfaction, with the predominant response that the work was overloaded with unnecessary administrative detail and that the job was too stressful. Moreover, GPs demonstrated little agreement that there was a good balance between effort and reward in work. Factors associated with the individual work satisfaction variables were the estimated practice population size, working hours, and age. Higher values for these variables were associated with higher self-reported stress and higher perception of unnecessary administrative duties. Surprisingly, although they perceived high stress and poor balance between effort and reward, Austrian GPs acknowledged a preserved interest for their work. This might demonstrate that GPs already in practice do find value in their work, perhaps due to patient continuity, satisfaction in providing care, or opportunity to serve as a community leader, particularly in rural settings, despite increasing workloads. However, these are the physicians who have already been in practice and experienced shifting work patterns, but those who have yet to enter practice may view these burdens as reasons not to enter the field altogether.

Our findings are of high relevance for Austria and other countries with similar health care systems, and should be integrated into future programs on strengthening health care systems and the primary health care sector. Finally, our findings are supported by the literature, showing that a substantial change in the level of exposure of under- and postgraduate education in general practice can advance the perceived status and value of general practice (7,9,23-25,32-35).

One strength of this study is that it was the first in-depth analysis to assess the working situation of GPs in urban, intermediate, and rural areas in Austria. An additional strength is that the survey was conducted as part of the QUALICOPC study, using its standardized and well-developed questionnaire. Nevertheless, this study has some limitations. Participation by GPs was voluntary and the return rate was low, leading to possible selection bias and limited generalizability. However, such low return rate is a well-known problem in primary care research, with typical rates ranging from 5%-15% (36,37). The return rate for example was similar to 14.5% obtained by the Australian QUALICOPC team (36). Particularly in countries such as Austria, where GP research is marginalized and the academic recognition is low, it is problematic to involve GPs in research activities performed in their free-time. It is possible that physicians with an even heavier workload did not have the time to respond, thus leading to lower estimates of workload. In addition, we included only GPs with a valid email address. This could have led to a selection bias, meaning that older GPs or those less technology savvy may not have had the opportunity to participate. Based on current demographics in Austria, it is precisely this population of older GPs that most frequently experiences heavier workload, thus again leading to possible lower estimates of workload (20). However, the sample surveyed does reflect the national sample according age, sex, distribution between the federal states, and the proportion between solo- and group practices. The distribution of Austrian GPs in 2011 according to sex was 39% for female GPs, the mean age was 52.5 years, and the proportion for group practice was 8% (20). Another important limitation is that the study relied on physicians’ estimation of variables, resulting in a high variability of answers. For example, the coefficient for the estimated size of the practice population of 0.002, is surprisingly low, leading to the assumption that GPs’ estimates of their practice population were quite inaccurate. This inaccuracy could be exacerbated by the observation that many GPs in Austria do not have a fixed patient list. This variability made it extremely difficult to exclude outliers and could have influenced the results regarding the real effect of practice size on the objective workload. Additionally, this is a cross-sectional study, which cannot be used to determine casual relationships.

In conclusion, to ensure patient access and primary care distribution in the future, the high number of on-call duties in rural areas, as well as the very high number of out-of-office services, estimated practice population, and patient contacts per day should be considered. We believe that the impact of these factors may be a reason for the declining interest in working as a GP in Austria. This study emphasizes the need for incentives that would motivate physicians to work as GPs. This is essential if we want to preserve the best care and access for Austrian patients, and ensure adequate physician distribution.
